# Perioperative pregabalin does not alter behavioural or diffuse noxious inhibitory control responses in 2 rat models of chronic pain

**DOI:** 10.1097/PR9.0000000000001450

**Published:** 2026-06-12

**Authors:** Francesca Di Domenico, Mateusz W. Kucharczyk, Ryan Patel, Kirsty Bannister

**Affiliations:** a UCL, Cell and Developmental Biology, London, United Kingdom; b Polish Centre for Technology Development, Łukasiewicz-PORT, Wrocław, Poland; c Department of Life Sciences, Imperial College London, South Kensington Campus, London, United Kingdom

**Keywords:** Perioperative analgesia, Refinement, Neuropathy, Cancer-induced bone pain, Behaviour

## Abstract

**Introduction:**

Multiple mechanisms contribute to the experience of pain where the use of model organisms to dissect mechanistically sensory regulatory circuitry is a vital component of discovering underlying causes of persistent pain in disease states. Such disease states can be modelled in animals using surgical procedures that, ethically, should involve administration of analgesia. However, since basic pain researchers often wish to measure pain-related events, animals may be denied perioperative analgesia to avoid adversely influencing experimental outcomes.

**Methods:**

We conducted a structured review of perioperative analgesia usage in rat spinal nerve ligation (SNL) and cancer-induced bone pain (CIBP) models. Using a combination of behavioural testing and in vivo electrophysiology in the dorsal horn of the spinal cord, we assessed the impact of perioperative pregabalin on nociceptive behaviours in the acute recovery phase, and behavioural and electrophysiological experimental outcomes in the established phase, of rat SNL and CIBP models.

**Results:**

A literature search revealed that, for studies using rat models of SNL or CIBP, only 5.37% and 12.69%, respectively, reported the use of perioperative analgesia. We then demonstrated that the use of pregabalin as a perioperative analgesic reduced mechanical hypersensitivity in the acute period after SNL surgery, with no impact on behavioural, electrophysiological, or neuropharmacological outcomes in the established phase of either model.

**Conclusions:**

This study challenges the basic science researcher's reasoning that perioperative analgesia confounds neurobiological outcomes. The use of perioperative analgesia should be an important consideration to improve animal welfare in chronic models of pain.

## Abstract 2

Perioperative analgesia can improve animal welfare without adversely affecting long-term behavioural or pharmacological experimental outcomes in chronic pain models.

## 1. Introduction

It is estimated that more than 100 million animals are used in research laboratories around the world each year. Animals involved in scientific procedures frequently experience varying degrees of pain and distress, with procedures classified according to severity. In accordance with UK regulations, researchers must implement strategies to minimise pain and discomfort in experimental protocols. Adequate perioperative care, including appropriate anaesthetics and analgesics during surgical procedures, is essential in mitigating pain and distress in laboratory animals.^19^

In chronic pain models, establishing a persistent pain state is crucial for disease modelling. However, despite the well-documented benefits of perioperative analgesia, its administration remains inconsistently implemented in the field.^8^ Although researchers can often anticipate the type and severity of pain experienced by experimental animals undergoing surgical procedures, concerns about the potential impact of analgesic medications on outcome measures may discourage the implementation of pain-relief regimens.^9,11,19^ However, identifying the specific reasons for the inconsistent use of perioperative analgesia is challenging, as they vary. Contributing factors may include a reluctance to modify well-established protocols because of concerns about compromising research outcomes, particularly relevant for principal investigators aiming to replicate previous research.

Clinically, perioperative analgesia is routinely administered to alleviate postsurgical pain. Over the past decade, pregabalin has gained attention^16,23^; perioperatively, it reduces both pain and opioid consumption during the acute postoperative period, with effects observed up to 48 and 72 hours after thoracotomy^10^ and lower limb orthopaedic surgery.^6^

Here, we review the reporting of perioperative analgesic use in 2 commonly used rat models of chronic pain, the spinal nerve ligation (SNL) model of neuropathy and cancer-induced bone pain (CIBP). We additionally investigate whether administering perioperative pregabalin to SNL and CIBP rats (1) improves behavioural hypersensitivity in the acute postoperative phase and (2) affects experimental outcome measures (behavioural hypersensitivity, neuronal excitability, and descending inhibitory signalling) in the established phase of the models. To this end, we used 2 reference datasets, which did not deliver perioperative analgesia, as an experimental framework, and we conducted a replicatory study aimed at confirming pathophysiological changes in the function of descending inhibitory signalling in SNL and CIBP rats. As gabapentinoids can exert analgesic effects through multiple peripheral and central mechanisms^3,4,18^ including disinhibiting locus coeruleus neurons to increase descending inhibition,^12,20,21^ we investigated whether perioperative pregabalin negatively affected the expression of these particular pathophysiological mechanisms.

## 2. Methods

### 2.1. Database search methods

A narrative review of the literature was conducted to identify original research articles investigating analgesia and pain outcomes in rodent models. A structured search was performed using PubMed (sole database). The search included publications from 1976 to 2022. Search terms captured studies addressing relevant species, interventions, and outcomes. Keywords included: ((rat OR rats OR murine OR mouse OR mice) AND (analgesic OR analgesia OR “spinal nerve ligation” OR SNL OR “cancer induced bone pain” OR “bone cancer pain”) AND (behavior* OR “pain behavior” OR “nociceptive behavior” OR “in vivo electrophysiology” OR electrophysiology OR “calcium imaging”)) NOT (review[Publication Type] OR editorial[Publication Type] OR letter[Publication Type] OR case reports[Publication Type]).

Eligibility criteria: (1) original research articles published in peer-reviewed journals, (2) studies that included an assessment of pain, (3) studies using rodent models of spinal nerve ligation or cancer-induced bone pain, and (4) studies that included at least one behavioural measure of pain.

Exclusion criteria: dissertations, books, book chapters, editorials, letters, case studies, conference proceedings, and abstracts. Narrative and systematic reviews were not included as primary sources; however, they were screened to identify additional relevant original research articles. Studies were also excluded if they used species other than rats (only rat studies were included), investigated neuropathic pain models other than spinal nerve ligation, and/or did not include a behavioural outcome measure of pain.

Titles and abstracts retrieved from the search were screened for relevance according to the inclusion/exclusion criteria and full texts were then assessed to determine final inclusion. Reference lists of relevant narrative and systematic reviews were also examined to identify additional original research articles that met the eligibility criteria.

### 2.2. Animals

Adult male Sprague-Dawley rats (126 in total) were used for all experiments (Charles River, Massechusetts USA). Animals were group housed (maximum of 5) on a conventional 12:12 hours light–dark cycle; food and water were available ad libitum. Temperature (20–22°C) and humidity (55%–65%) of holding rooms were closely regulated. Experimental design/analysis was conducted according to ARRIVE guidelines. All procedures were approved by the UK Home Office (licence PP0933098) under the Animals (Scientific Procedures, California, USA) Act 1986. Only male animals were used to make direct comparisons with reference datasets.^2,14^

### 2.3. Cancer-induced bone pain model

Cancer-induced bone pain was induced as described previously.^14^ Briefly, rats weighing 120 to 140 g were anaesthetised using isoflurane (induction 3% vol/vol, maintenance 1.5%–2% vol/vol) delivered in oxygen (1 L/min). Under aseptic conditions, a small incision was made over the right tibia anterior-medial surface and the bone exposed. Using a dental drill, a hole was made in the tibia to allow for thin polyethylene tube insertion 1 to 1.5 cm into the intramedullary cavity. On surgery day, flask adherent MRMT-1 cells were released from the flask surface by brief exposure to 0.1% wt/vol trypsin-ethylenediaminetetraacetic acid and subsequently pelleted by centrifugation (5 minutes, 1000 rpm). The pellet was washed with Hanks balanced salt solution (HBSS) without calcium, magnesium, or phenol red (ThermoFisher, Paisley, United Kingdom—cat. no. 14175095) and centrifuged again (5 minutes, 1000 rpm). The MRMT1 cell pellet was suspended in HBSS to a final concentration of 300,000 cells/mL (live cells counted using Tryptan Blue staining, Sigma, Missouri, USA) and kept on ice until use. After each surgery, cell viability was monitored, revealing no more than 10% cell death after ice storage (approximately 4 hours duration). Using a Hamilton syringe, 3,000 MRMT-1 carcinoma cells in 10 μL HBSS or 10 μL HBSS alone (to generate a sham model) was injected into the cavity. The tube was removed, and the hole plugged with bone restorative material. The surrounding skin was closed with absorbable 4-0 sutures. Rats were given an intraperitoneal injection of either 3 mg/kg pregabalin (PGB; gift from Pfizer, synthesised in-house) or vehicle (VEH; normal saline) before being allowed to recover in a temperature-controlled chamber. The experimenter was blinded to the injection material (MRMT-1 cells or HBSS) during surgery and perioperative analgesia.

### 2.4. Spinal nerve ligation model

Spinal nerve ligation was performed as described previously.^2^ Briefly, rats weighing 120 to 140 g were anaesthetised using isoflurane (induction 3% vol/vol, maintenance 1.5%–2% vol/vol) delivered in oxygen (1 L/min). Under aseptic conditions, a paraspinal incision was made and the tail muscle retracted from the spinal column. Part of the L5 transverse process was removed to expose the left L5 and L6 spinal nerves, which were then isolated with a glass nerve hook and ligated with a nonabsorbable 6-0 braided silk thread proximal to the formation of the sciatic nerve. The surrounding skin and muscle was closed with absorbable 4-0 sutures. Sham surgery was performed in an identical manner omitting the nerve isolation and ligation step. Rats were given an intraperitoneal injection of either 3 mg/kg pregabalin (PGB; gift from Pfizer, synthesised in-house) or vehicle (VEH; normal saline) before being allowed to recover in a temperature-controlled chamber.

### 2.5. Behavioural tests

All behavioural assessments were conducted by a single experimenter at a consistent time of day during the light phase. Animals were allowed to habituate to handling, the experimenter and apparatus before all behavioural testing. For assessment of mechanical withdrawal thresholds, after room acclimatisation (1 hours), rats were placed in isolation inside Perspex chambers on a wire mesh floor and left to acclimatise for a further 15 minutes. Mechanical sensitivity was assessed using von Frey filaments (Touch-Test, North Coast Medical California, USA). Filaments were applied to the plantar hind paw of the rat until they buckled for 5 to 6 seconds. Lifting, flinching, and shaking were considered positive responses. Fifty percent withdrawal thresholds were determined using the up-down method described previously,^5^ using von Frey filaments with bending forces of 1.4 g, 2 g, 4 g, 6 g, 8 g, 10 g, and 15 g. Testing began at 4 g followed by the next weight up or down depending on a negative or positive response, respectively. After a change in direction of the response a further 4 filaments, with a 2-minute interstimulus recovery period between each stimulation. Paw withdrawal thresholds (50% PWT) were calculated with the following formula: 50% PWT = (10^(x + kδ)^/10,000), where x represents the log of the last von Frey tested, and δ represents the mean difference between the von Frey filaments in log units (0.17) and k, a value dependent on the series of responses.

Weight bearing was assessed using an incapacitance tester (Linton Instrumentation, Norfolk, United Kingdom) in which rats were placed in a plexiglass enclosure where each hind paw resides on a separate weighing plate. Once the animal acquired a relaxed position, the mass exerted by each hind paw was measured 5 times (expressed in grams), with a resting period between measurements to allow the animal to reequilibrate or slightly shift weight distribution (around 10–20 seconds). Measurements from each paw were averaged separately, and results were transformed to give the percentage of weight borne on each side to the total rear leg bearing (taken as 100%).

### 2.6. In vivo electrophysiology

In vivo electrophysiology was performed days 14 to 15 post-CIBP induction and days 10 to 14 post-SNL induction (in 250–300 g rats). Anaesthesia was initially induced with 3.5% vol/vol isoflurane delivered in 3:2 ratio of nitrous oxide and oxygen. Once areflexic, a tracheotomy/canulation was performed, and rats were subsequently maintained on 1.5% vol/vol isoflurane for the remainder of the experiment (approximately 3–4 hours; core body temperature was maintained throughout with the use of a homeothermic blanket). Rats were secured in a stereotaxic frame, and a laminectomy was performed to expose the L4-L6 segments of the spinal cord; 2 spinal clamps were applied to stabilise the spinal column. Extracellular recordings were obtained from deep dorsal horn wide dynamic range lamina V/VI neurones with receptive fields on the glabrous skin of the hind toes using 127-µm diameter 2-MΩ parylene-coated tungsten electrodes (A-M Systems, Sequim, WA). The search stimulus consisted of light tapping of the hind paw as the electrode was manually lowered. Neurones were characterised from depths relating to the deep dorsal horn laminae, and once a single unit was isolated, neurones were classified as wide dynamic range based on sensitivity to dynamic brushing, noxious mechanical (60 g) and noxious heat stimulation (48°C) of the receptive field. Data were captured and analysed by a CED Micro1401 interface coupled to a computer with Spike2 v4 software (Cambridge Electronic Design, Cambridge, United Kingdom). The signal was amplified (preamp + amp ×30–40 k), bandpass filtered (low/high frequency cut-off 1/3 kHz), and digitised at rate of 20 kHz. A HumBug (Quest Scientific, British Columbia, Canada) was used to remove low frequency noise (50–60 Hz).

Each baseline trial involved applying von Frey filaments (8 g, 26 g, and 60 g) to the receptive field for 10 seconds per stimulus (60 seconds recovery period between stimuli). Evoked responses were quantified as the total number of neuronal events during the 10-second stimulation. Once stable neuronal responses were obtained (3 consistent recordings <10% variation in response), a diffuse noxious inhibitory controls (DNIC) test was applied. Diffuse noxious inhibitory controls were activated by a noxious clamp (a 35-mm bulldog serrefine [Interfocus, Linton, United Kingdom—Cat. No. 18050-28]) applied to the ipsilateral ear concurrently to stimulation of the hind paw with von Frey filaments. Expression of DNIC was defined as a decrease in evoked response (>10% from baseline) during the application of a conditioning stimulus as per our previous investigations.^1,2,15^ A 10-minute nonstimulation recovery period was allowed before 2 more baseline trials were conducted (baseline responses were calculated as a mean of 3 baseline trials). Atipamezole (100 μg/50 μL [Tocris, Abingdon, United Kingdom—cat. no. 2937]; vehicle: 97% normal saline/2% cremophor/1% DMSO Sigma-Aldrich, Gillingham, United Kingdom—cat. no. C5135, D5879) was subsequently applied topically to the spinal cord, and tests repeated at 10, 20, 30, and 60 minutes postdosing; peak change from baseline is plotted.

### 2.7. Statistics

Statistical analyses were performed using SPSS v29 (IBM, Armonk, NY). The experimental unit for behaviour and in vivo electrophysiology was the individual rat. Rats were pseudorandomised into experimental/treatment groups by an independent investigator using a random number generator. All behavioural experiments were conducted blind to experimental variables; unblinding was performed after data collection. Group sizes were determined by a priori calculations using the following assumptions: F test, α 0.05, 1-β 0.8, ε 1, effect size range *d* = 0.5 to 0.8 for electrophysiological measures, effect size range *d* = 0.3 to 0.5 for behavioural measures. Mechanical coding of neurones was compared with a 2-way repeated measures (RM) ANOVA, followed by a Bonferroni *post hoc* test for paired comparisons. Where appropriate, sphericity was tested using Mauchly test; the Greenhouse–Geisser correction was applied if violated. Main effect of DNIC or drug is reported throughout. Mechanical hypersensitivity and weight bearing were assessed with either a Friedman test or Mann–Whitney. Effect sizes are reported as Cohen *d*. All data plotted represent mean ± SEM, *P < 0.05, *P* < 0.01, ***P < 0.001.

## 3. Results

### 3.1. A review of the literature: the frequency of perioperative analgesic use in spinal nerve ligation and cancer-induced bone pain models

**Figure 1.**
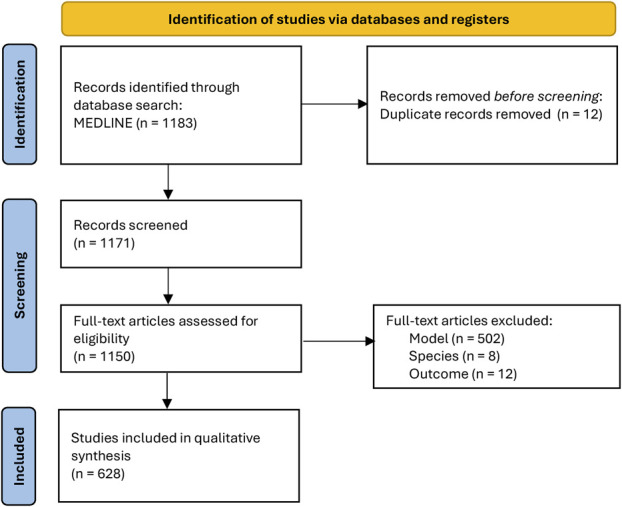
PRISMA flow diagram. This diagram shows the systematic process we followed to include papers captured by our search. Adapted from^17^ under a creative commons licence CC BY 4.0.

The search identified 1183 papers between 1976 and 2022 that cited the use of animals undergoing SNL or CIBP surgery (Fig. 1). For the SNL model, we found 1012 papers that cited the use of spinal nerve ligation, of which 498 papers were excluded from the study. Thus, information was collated from 502 studies (Table 1). The analysis revealed that of the 502 original research articles, only 5.37% (27 studies) reported the use of perioperative analgesia. Drugs targeting NMDA receptors were the most frequent choice (23 studies), followed by α2 adrenoceptor agonists (2 studies), opioids (1 study), Non steroidal anti-inflammatory drugs (NSAIDs) (1 study), and benzodiazepines (1 study). The remaining 475 did not report the use of perioperative analgesia. No studies explicitly stated, “no peri-operative analgesia was provided.”

**Table 1:** Details from papers that reported the use of perioperative analgesia in spinal nerve ligation and cancer-induced bone pain rats (number of papers).

Model	Papers included	Explicitly state “no perioperative analgesia provided”	No mention of perioperative analgesia	Use of perioperative analgesia	Perioperative analgesic provided							
					Meloxicam	Ketamine	Carprofen	Lidocaine	Buprenorphine	Zoletil	Midazolam/hypnorm	Medetomidine
SNL	502	0	474	27	1	21	0	0	1	2	1	2
CIBP	126	0	110	16	5	5	5	1	0	0	0	1

CIBP, cancer-induced bone pain; SNL, spinal nerve ligation.

We found 171 papers that cited the use of a rodent model of CIBP, of which 45 papers were excluded from the study. Thus, information was collated from 126 studies (Table 1). The analysis revealed that of the 126 original research articles, only 12.69% (16 studies) reported the use of perioperative analgesia. NSAIDs were the most frequent choice (10 studies), followed by drugs targeting NMDA receptors (5 studies) and local anaesthetics (1 study). The remaining 113 articles did not report the use of perioperative analgesia and no studies explicitly stated, “no peri-operative analgesia was provided.”

### 3.2. Spinal nerve ligation, but not cancer-induced bone pain rats, showed a mild improvement in mechanical hypersensitivity in the acute postsurgical phase when pregabalin was administered as a perioperative analgesic

**Figure 2.**
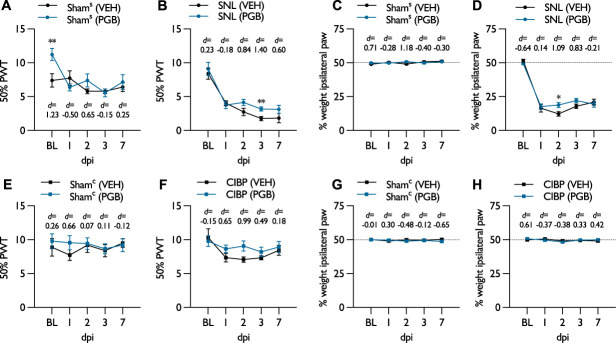
Postsurgical outcomes: effect of perioperative pregabalin on mechanical hypersensitivity in SNL and CIBP rats during the acute postoperative phase. Development of mechanical hypersensitivity in (A) sham^s^ (*n*_*VEH*_ = 10, *n*_*PGB*_ = 12) and (B) SNL rats (*n*_*VEH*_ = 10, *n*_*PGB*_ = 12). Changes in weight bearing in (C) sham^s^ and (D) SNL rats. Development of mechanical hypersensitivity in (E) sham^c^ (*n*_*VEH*_ = 9, *n*_*PGB*_ = 9) and (F) CIBP rats (*n*_*VEH*_ = 10, *n*_*PGB*_ = 12). Changes in weight bearing in (G) sham^c^ and (H) CIBP rats. Data represent mean ± SEM; *P < 0.05, **P < 0.01 (Mann–Whitney test). Asterisks and Cohen *d* values denote difference between vehicle- and pregabalin-treated groups. BL, baseline; CIBP, cancer-induced bone pain; dpi, days postinjury; PGB, pregabalin; SNL, spinal nerve ligation; VEH, vehicle.

We examined the effect of perioperative pregabalin on the development of mechanical hypersensitivity during the acute postoperative phase defined as days 1 to 7 postinjury; sham animals (sham^s^ and sham^c^ respectively) were included as control groups, which experienced acute tissue damage but no chronic injury. There was no change from baseline of mechanical withdrawal thresholds in the sham^s^ group given perioperative vehicle, but we observed a decrease in the pregabalin group (Friedman test: sham^s^ [VEH] *P* = 0.135, sham^s^ [PGB] *P* = 0.00003) (Fig. 2A). Both SNL groups rapidly developed mechanical hypersensitivity from day 1 onwards (Friedman test: SNL [VEH] *P* = 0.00002, SNL [PGB] *P* = 0.000005) (Fig. 2B), with evidence of higher withdrawal thresholds in SNL rats receiving perioperative analgesia on day 3 postinjury (Mann–Whitney, day 2: *P* = 0.091, day 3: *P* = 0.00054, day 7: *P* = 0.088). Sham^s^ rats treated with pregabalin did not exhibit any time-dependent changes in weight bearing with weak evidence for altered weight bearing observed in vehicle-treated rats (Friedman test: sham^s^ [VEH] *P* = 0.022, sham^s^ [PGB] *P* = 0.52) (Fig. 2C). Both SNL groups avoided placing weight on the injured paw from day 1 onwards (Friedman test: SNL [VEH] *P* = 0.00006, SNL [PGB] *P* = 0.00003) (Fig. 2D), with evidence of greater avoidance in SNL rats not receiving perioperative analgesia on day 2 postinjury (Mann–Whitney, day 2: *P* = 0.021, day 3: *P* = 0.15).

There was no change from baseline of mechanical withdrawal thresholds in sham^c^ groups given perioperative vehicle or pregabalin (Friedman test: sham^c^ [VEH] *P* = 0.43, sham^c^ [PGB] *P* = 0.93) (Fig. 2E). Neither CIBP groups developed mechanical hypersensitivity (Friedman test: CIBP [VEH] *P* = 0.12, CIBP [PGB] *P* = 0.42) (Fig. 2F). Weight bearing was not altered in sham^c^ (Friedman test: sham^c^ [VEH] *P* = 0.32, sham^c^ [PGB] *P* = 0.45) (Fig. 2G) or CIBP rats (Friedman test: CIBP [VEH] *P* = 0.25, CIBP [PGB] *P* = 0.21) (Fig. 2H). Heat hypersensitivity was not observed in either SNL or CIBP rats (data not shown).

### 3.3. Administration of perioperative pregabalin does not adversely affect nocifensive behaviours in the established phase of spinal nerve ligation and cancer-induced bone pain models

**Figure 3.**
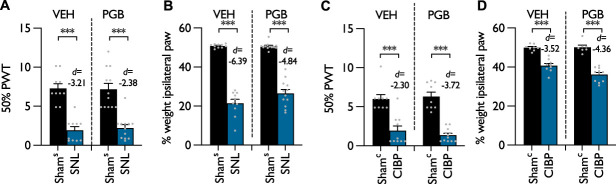
Experimental outcomes (*i*): effect of perioperative pregabalin on the establishment of chronic pain in SNL and CIBP rats. (A) Effect of perioperative pregabalin on mechanical hypersensitivity in sham^s^ (*n*_*VEH*_ = 10, *n*_*PGB*_ = 12) and SNL rats (*n*_*VEH*_ = 10, *n*_*PGB*_ = 12) on day 14 postinjury. (B) Effect of perioperative pregabalin on weight bearing in sham^s^ and SNL rats on day 14 postinjury. (C) Effect of perioperative pregabalin on mechanical hypersensitivity in sham^c^ (*n*_*VEH*_ = 9, *n*_*PGB*_ = 9) and CIBP rats (*n*_*VEH*_ = 10, *n*_*PGB*_ = 12) on day 14 postinjury. (D) Effect of perioperative pregabalin on weight bearing in sham^c^ and CIBP rats on day 14 postinjury. Data represent mean ± SEM; ***P < 0.001 (Mann–Whitney test). Asterisks and Cohen *d* values denote difference between sham and SNL/CIBP groups, CIBP, cancer-induced bone pain; PGB, pregabalin; SNL, spinal nerve ligation; VEH, vehicle.

Having examined the effect of perioperative pregabalin on the development of nocifensive behaviours in the acute (immediate) postoperative phase (Fig. 2), we next examined whether administering perioperative analgesia affected behavioural experimental outcomes in the established phase of the models. At day 14 postinjury, SNL rats developed mechanical hypersensitivity (Mann–Whitney, VEH: *P* = 0.00003, PGB: *P* = 0.000005) (Fig. 3A) and exhibited altered weight bearing (Mann–Whitney, VEH: *P* = 0.00001, PGB: *P* = 0.000001) (Fig. 3B); however, no difference in the magnitude of mechanical hypersensitivity or weight bearing was observed between rats receiving perioperative vehicle or pregabalin. Similarly, CIBP rats developed mechanical hypersensitivity (Mann–Whitney, VEH: *P* = 0.0005, PGB: *P* = 0.000007) (Fig. 3C) and exhibited altered weight bearing (Mann–Whitney, VEH: *P* = 0.00008, PGB: *P* = 0.00006) (Fig. 3D); however, no difference in the magnitude of mechanical hypersensitivity or weight bearing was observed between rats receiving perioperative vehicle or pregabalin.

### 3.4. Administration of perioperative pregabalin does not affect the expression of descending inhibition in spinal nerve ligation and cancer-induced bone pain rats

**Figure 4.**
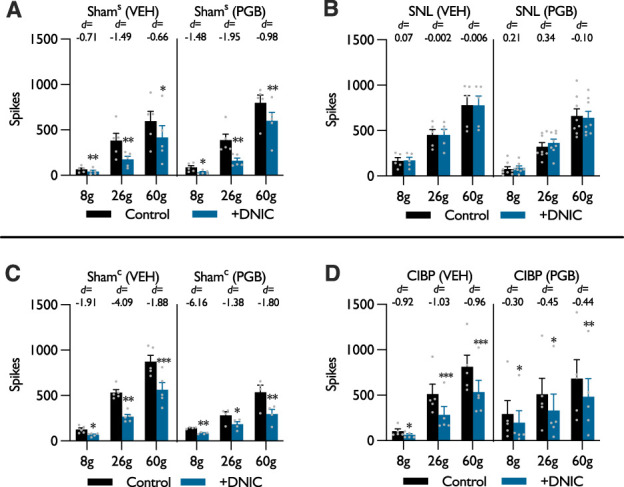
Experimental outcomes (*ii*): effect of perioperative pregabalin on the expression DNIC in SNL and CIBP rats. Effect of activating DNIC on neuronal responses to mechanical stimulation in (A) sham^s^ (*n*_*VEH*_ = 5, *n*_*PGB*_ = 5) and (B) SNL rats (*n*_*VEH*_ = 5, *n*_*PGB*_ = 8). Effect of activating DNIC on neuronal responses to mechanical stimulation in (C) sham^c^ (*n*_*VEH*_ = 5, *n*_*PGB*_ = 4) and (D) CIBP rats (*n*_*VEH*_ = 5, *n*_*PGB*_ = 5). Data represent mean ± SEM; *P < 0.05, **P < 0.01, ***P < 0.001 (2-way RM ANOVA with Bonferroni post hoc). Asterisks and Cohen *d* values denote difference between control and DNIC groups. CIBP, cancer-induced bone pain; DNIC, diffuse noxious inhibitory controls; PGB, pregabalin; SNL, spinal nerve ligation; VEH, vehicle.

We examined whether administering perioperative pregabalin affected endogenous pain modulation in the established phase of the models. In vivo electrophysiology was performed in the dorsal horn of the spinal cord to record from wide dynamic range neurones in lamina V/VI. First, descending inhibition was assessed by activating DNIC, which were recruited by applying a distant noxious stimulus during hind paw stimulation and are subserved by noradrenergic signalling.^2,15^ Diffuse noxious inhibitory controls were expressed in sham^s^ rats in perioperative vehicle- (2-way RM ANOVA: *P* = 0.02, *F*_1,4_ = 50.952) and pregabalin-treated groups (2-way RM ANOVA: *P* = 0.000007, *F*_1,4_ = 286.94) (Fig. 4A). Diffuse noxious inhibitory controls were absent in both perioperative vehicle- and pregabalin-treated SNL rats (2-way RM ANOVA: VEH—*P* = 0.804, *F*_1,4_ = 0.07; PGB—*P* = 0.451, *F*_1,7_ = 0.636) (Fig. 4B). Diffuse noxious inhibitory controls were expressed in sham^c^ rats in perioperative vehicle- (2-way RM ANOVA: *P* = 0.001, *F*_1,4_ = 74.752) and pregabalin-treated groups (2-way RM ANOVA: *P* = 0.0034, *F*_1,3_ = 72.077) (Fig. 4C). Diffuse noxious inhibitory controls were expressed in CIBP rats in perioperative vehicle- (2-way RM ANOVA: *P* = 0.0002, *F*_1,4_ = 172.075) and pregabalin-treated groups (2-way RM ANOVA: *P* = 0.0032, *F*_1,4_ = 39.95) (Fig. 4D).

**Figure 5.**
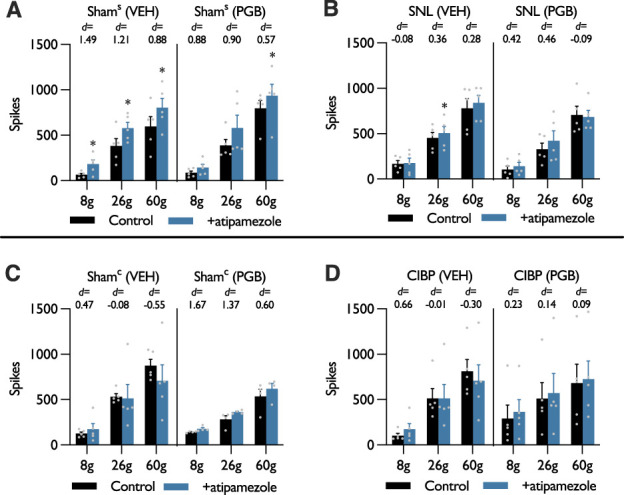
Experimental outcomes (*iii*): effect of perioperative pregabalin on noradrenergic inhibitory tone in SNL and CIBP rats. Effect of spinal atipamezole on neuronal responses to mechanical stimulation in (A) sham^s^ (*n*_*VEH*_ = 5, *n*_*PGB*_ = 5) and (B) SNL rats (*n*_*VEH*_ = 5, *n*_*PGB*_ = 5). Effect of spinal atipamezole on neuronal responses to mechanical stimulation in (C) sham^c^ (*n*_*VEH*_ = 5, *n*_*PGB*_ = 4) and (D) CIBP rats (*n*_*VEH*_ = 5, *n*_*PGB*_ = 5). Data represent mean ± SEM; ***P < 0.001 (2-way RM ANOVA with Bonferroni post hoc). Asterisks and Cohen *d* values denote difference between control and atipamezole groups. CIBP, cancer-induced bone pain; PGB, pregabalin; SNL, spinal nerve ligation; VEH, vehicle.

Subsequently, noradrenergic inhibitory tone was revealed by applying the α_2_-adrenoceptor antagonist atipamezole topically to the spinal cord; an increase in firing frequency to evoked stimuli reflects the presence of descending inhibitory signalling. Neuronal responses to mechanical stimulation were facilitated in sham^s^ rats in perioperative vehicle- (2-way RM ANOVA: *P* = 0.022, *F*_1,4_ = 13.168) and pregabalin-treated groups (2-way RM ANOVA: *P* = 0.0495, *F*_1,4_ = 7.762) (Fig. 5A). Compared with sham^s^ rats, inhibitory tone was reduced in SNL rats in both the perioperative vehicle- (2-way RM ANOVA: *P* = 0.001, *F*_1,4_ = 70.259) and pregabalin-treated groups (2-way RM ANOVA: *P* = 0.730, *F*_1,4_ = 0.137) (Fig. 5B). Neuronal responses were unaffected in sham^c^ rats after atipamezole dosing in groups treated with perioperative vehicle (2-way RM ANOVA: *P* = 0.751, *F*_1,4_ = 0.115) and pregabalin (2-way RM ANOVA: *P* = 0.08, *F*_1,3_ = 6.775) (Fig. 5C). Neuronal responses in CIBP rats were unaffected postdosing of atipamezole in the perioperative vehicle-treated group (2-way RM ANOVA: *P* = 0.815, *F*_1,4_ = 0.63), but weak evidence was found for altered neuronal responses in the pregabalin-treated group (2-way RM ANOVA: *P* = 0.005, *F*_1,4_ = 32.672, paired comparisons *P* > 0.05) (Fig. 5D). Effect sizes were broadly comparable across all groups.

## 4. Discussion

A significant proportion of published original research articles using rats undergoing surgery for spinal nerve ligation or cancer-induced bone pain models do not cite the use/administration of perioperative analgesia. This was unexpected—in the United Kingdom alone, the use of analgesics during surgery is deemed necessary for moderate to severe procedures by regulatory bodies. It is noteworthy that the umbrella of dates captured by our search began as early as 1976. One would expect that, proportionally, the number of studies citing the use of perioperative analgesics will increase as time goes on, particularly in countries where animal welfare is governed and/or informed by bodies such as the NC3Rs. Nonetheless, our findings demonstrate that historically as well as recently, animal welfare is not always reported in research papers for a significant number of animals undergoing surgeries to induce a chronic pain state.

In our study, pregabalin was chosen as the perioperative analgesic for several reasons. First, because of its use clinically as an opioid replacement during surgical procedures,^7^ second because of its known peripheral and spinal mechanism of action in neuropathic pain,^4,18^ and third because of its modulation of affective-motivational qualities of pain.^3^ A key consideration for interpreting the behavioural data is the application of tests to the hind paw in both models. For the SNL model, this is the injured territory of the nerve and represents primary mechanical hypersensitivity, whereas for the CIBP model, this represents secondary mechanical hypersensitivity, which develops on a longer timescale in a manner that tracks with trabecular bone destruction.^13,14^ We did not observe secondary hypersensitivity in the CIBP model during the acute postoperative phase, and pregabalin did not affect the development of secondary mechanical hypersensitivity at day 14 of the model. In contrast, primary mechanical hypersensitivity was evident at day 1 postinjury in the SNL model and perioperative pregabalin provided mild relief on days 2 and 3 postinjury without affecting behavioural responses in the established phase of the model. Collectively, these observations suggest that perioperative pregabalin improves primary pain after injury without affecting the establishment of primary or secondary hypersensitivity in the established phase. With a single dose, the precise mechanism of action of perioperative pregabalin is unclear. Chronic dosing is known to reduce peripheral axonal trafficking of α2δ-1 subunits,^4^ but this mechanism cannot account for the effects observed here, which are suggestive of an acute/transient central mechanism of action possibly including actions within noradrenergic systems.^12,20,21^

After demonstrating that behavioural outcomes were not affected by perioperative analgesia, we replicated 2 previous electrophysiological studies of descending inhibition in the SNL and CIBP models in male rats. These previous studies did not administer perioperative analgesics allowing us to draw comparisons with these reference datasets. First, we show here that the expression of an endogenous modulatory circuit, DNIC, was not affected by perioperative pregabalin. We have previously observed an absence of DNIC in the SNL model^2^ and preservation of DNIC in the CIBP model^14^ at comparable time points. Second, we investigated tonic noradrenergic inhibitory tone and observed that the effects of spinal atipamezole on neuronal excitability was consistent between perioperative vehicle and pregabalin groups, and in line with our previous observations.^2,14^ These data indicate that perioperative pregabalin does not adversely affect this pathophysiological mechanism nor the functionality of endogenous pain modulatory pathways in the established phase of SNL and CIBP models. A limitation of our study was the use of male rats only; we have previously observed no sex differences in DNIC expression or the effect of spinal atipamezole on neuronal excitability in sham and CIBP rats.^14,22^ We cannot rule out sex differences in pregabalin efficacy.

In summary, our data support that perioperative analgesia can improve animal welfare in the acute postoperative phase without affecting desirable outcomes in the established phase. Developing a standardised framework for perioperative analgesia is challenging and must be adapted according to the experimental objectives. For example, researchers investigating the development of a chronic pain state would be negatively affected by perioperative analgesia. However, most studies are based on an established chronic pain state to reflect a clinical situation. Perioperative analgesic regimens for chronic pain models should be explored widely and factored into experimental design as refinement of models is possible without detriment to studying pathophysiological mechanisms, contrary to historical dogma.

## Disclosures

The authors have no conflict of interest to declare.
